# Intranasal Mupirocin to Reduce Surgical Site Infection Post Cardiac Surgery: A Review of the Literature

**DOI:** 10.7759/cureus.33678

**Published:** 2023-01-12

**Authors:** Jessy Nellipudi, Caleb Stone

**Affiliations:** 1 General Surgery, Mount Isa Hospital, Mount Isa, AUS; 2 Surgery, Princess Alexandra Hospital, Brisbane, AUS

**Keywords:** prophylactic antibiotics, staphylococcus aureu, sternal wound infection, mupirocin, cardiac surgery

## Abstract

Sternal wound infections after cardiac surgery are associated with high rates of morbidity and mortality. One of the known risk factors of sternal wound infection is Staphylococcus aureus colonisation. Intranasal mupirocin decolonisation therapy implemented pre-operatively appears to be an effective preventative measure in reducing sternal wound infections post-cardiac surgery. Therefore, the main aim of this review is to evaluate the current literature regarding the use of intranasal mupirocin before cardiac surgery and its impact on sternal wound infection rates.

## Introduction and background

Surgical site infection (SSI) is a concerning complication after surgery and has been associated with high rates of morbidity and mortality. The literature reports a highly variable incidence of SSI from 1% to 33%, with mortality rates as high as 47% in some cohorts [1-5]. Patients who experience SSI are more likely to report increased pain, less mobilisation, and increased hospital stay post-operatively [6]. SSIs are also associated with a heavy economic burden due to the increased length of hospital stay, readmissions, need for multiple investigations and treatment options, ICU bed requirement and cost of wound care materials. One of the most important SSIs after cardiac surgery is sternal wound infection (SWI). The annual burden of SWI post-cardiac surgery on the Australian healthcare system was recently estimated to be 9.2 million AUD [7]. To address this issue, mupirocin decolonisation of Staphylococcus aureus (S. aureus) has been described in the literature as an attractive treatment option to minimise sternal wound infection after cardiac surgery. Therefore, the main aim of this review is to evaluate the current literature regarding the use of intranasal mupirocin before cardiac surgery and its impact on sternal wound infection rates.

## Review

Sternal wound infection after cardiac surgery

Sternal wound infection can be classified as either superficial or deep. Superficial infections involve the skin and subcutaneous tissue layers and the pectoralis fascia, whereas deep infections involve the muscle layers, sternal bone and may involve deep-seated retrosternal tissue and mediastinal fascia. Rarely deep sternal wound infections progress to mediastinitis and affect the thoracic organs [6]. The incidence of superficial SWI ranges between 0.5-8%. The incidence of deep sternal wound infection ranges between 0.6-5%, with an in-hospital mortality rate between 7-47% [8, 9]. The risk factors implicated in SWI have been widely studied to account for these alarmingly high infection rates. It is well-documented that obesity, smoking, diabetes and poor nutrition impair wound repair and play a major role in infection and inflammation [6]. Other risk factors associated with SWI are prolonged operative time, re-operation for bleeding, the presence of greater than three anastomoses and nasal carriage of S. aureus [10-13]. Once deep infection is established, the one-year post-operative mortality can be up to 20% which is substantially linked to concomitant morbidities such as sepsis, organ failure and gastrointestinal bleeding [11, 14].

Microbiology of sternal wound infection

The pathogens commonly isolated in sternal wound infections are Methicillin-sensitive (MSSA) and Methicillin-resistant Staphylococcus aureus (MRSA), coagulase-negative staphylococci (especially Staphylococcus epidermidis) and gram-negative bacteria [13, 15]. It is currently estimated that up to 75% of Staphylococcus epidermidis strains are methicillin-resistant [16]. The majority of SWI after cardiac surgery are caused by Gram-positive bacteria, followed by Gram-negative bacteria. Staphylococcus aureus has been implicated in up to half of SSI post-cardiac surgery [17]. Superficial sternal wound infections are usually adequately treated with intravenous antibiotics and judicious wound care. However, despite advances in treatment and prevention, deep sternal wound infections remain a serious complication after cardiac surgery. They may require prolonged treatment, return to theatre for debridement and contribute to excessive morbidity to patients post-cardiac surgery. Additionally, SWIs place an immense financial burden to the already strained health system.

Staphylococcus aureus

Staphylococcus aureus is a skin commensal organism that is estimated to colonise 16%-36% of the population. S. aureus can colonise different parts of the skin and mucosa. The main reservoir in humans is thought to be the nasal passages. Nasal colonisation by S. aureus is a well-documented risk factor for SWI [18]. Due to the rising number of MRSA infections among patients post-cardiac surgery and to reduce the incidence of SWI caused by S. aureus, nasal application of topical mupirocin has been suggested in the literature as a preventative intervention pre-operatively.

Intranasal mupirocin

Intranasal mupirocin ointment (Bactroban) is a topical antibiotic used to eradicate gram-positive and some gram-negative aerobes. Its main action is to inhibit bacterial protein synthesis. Historically, systemic antibiotics were ineffective in treating the nasal flora implicated in wound infections [19]. With the rate of nasal Staphylococcus aureus carriage at approximately 20-30% of the population and the number of Methicillin-resistant species on the rise, topical mupirocin has become the most effective treatment option in reducing Staphylococci colonisation [19, 20]. Mupirocin has demonstrated effective decolonisation of MSSA in >90% of cases and MRSA in about 50% of cases [21, 22].

Mupirocin in cardiac surgery

Given the morbidity and mortality associated with sternal wound infections post-cardiac surgery, any preventative strategies implemented to minimise the risk of sternal wound infections, particularly MRSA infections, would make a big difference to patient's overall post-operative course and experience. Various studies have evaluated the efficacy of intranasal mupirocin in minimising sternal wound infection in patients undergoing cardiothoracic surgery. Targeted mupirocin-based studies focus on decolonisation in carriers of S. aureus identified in pre-operative screening, whereas universal mupirocin bases studies focus on treating all patients irrespective of their carrier status.

Targeted mupirocin-based strategy

Concerning studies that focused on carriers of S. aureus, Bode et al. [23] conducted a large multi-centre randomised controlled trial of patients who underwent a surgical procedure (n=808) to evaluate if, in nasal carriers of S. aureus, treatment with mupirocin nasal ointment and chlorhexidine soap reduced the risk of hospital-associated S. aureus infection. In a subgroup analysis of this group, 391 patients underwent a cardiothoracic surgical procedure. In this group of patients who had treatment with intranasal mupirocin and chlorhexidine gluconate body wash, there was a statistically significant reduction of S. aureus surgical wound infection [1.4% vs 8.8%, relative risk (95% CI): 0.14 (0.01-0.51)] [23]. Important aspects to note in this study are that it was performed on all patients undergoing surgery and less than 50% comprised of cardiac surgery patients. Additionally, using simultaneous chlorhexidine to eliminate S. aureus from other external sources could have affected the study outcome, and furthermore, prophylactic antibiotics were also not standardised.

Additionally, various prospective and retrospective studies have shown varying levels of evidence. In a prospective study, Nicolas et al. [24] reported that patients who were successfully decolonised of S. aureus before undergoing cardiac surgery (using a combination of intra-nasal mupirocin, chlorhexidine shower and mouthwash) showed a non-significant trend towards reduced S. aureus surgical site infection (0% vs 10.5% p=0.06) [24]. While some large retrospective interrupted time series have shown that mupirocin-based decolonisation has reduced surgical site infection in patients who are S. aureus carriers [25, 26], other series have failed to show any difference in surgical site infection between groups [27, 28].

Universal mupirocin-based strategy

Universal mupirocin-based decolonisation has also been studied in various interrupted time series. These studies were usually performed in institutions where universal mupirocin base protocols were introduced without screening for carrier status before cardiac surgery.

Most recently Savary et al. [29], in a retrospective analysis of a paediatric population, showed that paediatric patients who underwent systemic decolonisation (2% intranasal mupirocin twice daily and daily skin wash with 0.5% chlorhexidine gluconate soap once daily for seven consecutive days before surgery) have significantly lower rates of S. aureus infection compared to the group which had no decolonisation (0.8% vs 5.7%, p<0.005).

Various other retrospective time series have also reported a significant reduction in overall sternal site infection and surgical site infections due to S. aureus infection after universal treatment with intranasal mupirocin. Usry et al. [30] have reported a 53% reduction in sternal surgical site infection among patients undergoing coronary artery bypass graft (CABG) who were treated with intranasal mupirocin (2.62% vs 1.24%, p=0.007). This was further supported by Cimochowski et al. [19], who reported a 33% decrease in sternal wound infections (2.7% vs 0.9%, p=0.005). This difference was consistent in both superficial and deep sternal wound infections. More recently, Lemaignen et al. [31] in a large series with 19 years of prospectively collected data, have shown that universal treatment with mupirocin and poly iodine body wash showed that the incidence of mediastinitis decreased significantly after initiation of S. aureus decolonisation (1.43% vs 0.61%, P < 0.001) [10].

Universal versus targeted decolonisation

From the available evidence, based primarily on retrospective analysis, it appears that intranasal mupirocin was effective at preventing surgical site infection when it was used in all patients, irrespective of their carrier status (universal decolonisation). Studies based on mupirocin use in carriers of S. aureus (targeted decolonisation) showed conflicting evidence. The randomised controlled trial conducted by Bode et al. demonstrated an overall reduction in sternal wound infection [23]. However, this study was not designed for cardiothoracic patients, as less than 50% of the sample represented cardiothoracic patients. Other studies focused on targeted therapy showed conflicting evidence. In general, there is a significant discrepancy in the studies with respect to study protocols, study cohorts and pre-operative antibiotic prophylaxis used. One important consideration is whether targeted decolonisation or universal decolonisation therapy should be used. Due to the discrepancy in the available data, the current guidelines vary in their recommendations. Theoretically, targeting decolonisation in carriers of S. aureus appears to be an attractive idea in patients undergoing cardiac surgery, as decolonisation therapy would not be useful in patients who are not carriers. However, the practicality and logistics of pre-operative testing, follow-up and administering treatment need to be considered when implementing a targeted decolonisation strategy and may be the reason why in some studies, universal decolonisation was more effective.

Limitations

There are important limitations to the available studies that need to be considered when interpreting and applying the available evidence. Firstly, a large number of studies are retrospective analyses that are subject to confounding, recall or misclassification bias. Secondly, a few studies used historical data as controls [19, 30, 31]; this can lead to various issues and biases with respect to collecting and maintaining data, differences in patient cohorts, hospital infection control protocols, operative technique, pre-operative antibiotic prophylaxis and changes in post-operative care over time which ultimately can affect outcomes concerning the control versus treatment groups. Furthermore, geographical differences may affect baseline colonisation rates of S. aureus. Finally, differences in patient population and risk factors may have influenced decolonisation rates and infection rates.

Mupirocin resistance

One of the main concerns, particularly with universal decolonisation, is the emergence of mupirocin resistance. However, the data is limited. The REDUCE MRSA trial [32], which compared targeted vs universal decolonisation with mupirocin, failed to show any difference in resistance between the two treatment arms. However, the authors concluded, “the confidence limits were broad, and the results should be interpreted with caution” [32]. Furthermore, a more recent meta-analysis conducted by Dadashi et al. [33] reports an increased prevalence of mupirocin resistance in both MSSA and MRSA. Further studies carefully designed to assess the impact of mupirocin resistance are required to evaluate the impact of mupirocin in the long term.

Cost analysis

Another important consideration in implementing mupirocin-based decolonisation is cost. Infection prevention strategies and programmes require high financial costs to establish and run effectively. Limited studies have reviewed the cost-effectiveness of mupirocin bases decolonisation therapies. In 2001, Cimochowski et al. [19] reviewed intranasal mupirocin and its cost-effectiveness. They reported a significant difference in sternal wound infection in the group treated with mupirocin (2.7% vs 0.9%; p=0.005). Further cost analysis revealed that a course of treatment with mupirocin was $12.47 USD compared to treatment of deep sternal wound infection which was $81,018 +/- $41,567 USD. They concluded that treatment with intranasal mupirocin was a safe and cost-effective method in reducing sternal wound infection. This was further supported by Hong et al. [17], who reported that compared to no decolonisation therapy, universal decolonisation decreased overall costs by $462 USD. In this study, universal decolonisation was a superior cost-saving strategy compared to no decolonisation and target decolonisation. This translated to a potential cost saving of $57 million per 220,000 coronary artery bypass graft procedures [17].

Current recommendations

Current recommendations vary concerning the type of mupirocin base strategy. The 2017 European Association of Cardio-Thoracic Surgery (EACTS) guidelines on perioperative medication recommend that in elective patients undergoing cardiac surgery, mupirocin twice daily intranasally is recommended four days before the procedure [34]. Whereas, the 2019 Guidelines for perioperative care in cardiac surgery recommendations by the Enhanced Recovery After Surgery Society recommend universal decolonisation with mupirocin [35]. Finally, in the expert consensus review published in the Journal of Thoracic and Cardiovascular Surgery on prevention and management of sternal wound infection, routine mupirocin administration was recommended in all cardiac surgery procedures in the absence of PCR testing or nasal cultures positive for S. aureus [36].

## Conclusions

In summary, due to the high burden of morbidity and mortality caused by sternal wound infections and the impact it has on patient's quality of life and the economic burden, the intra-nasal mupirocin decolonisation strategy appears to be promising in reducing sternal wound infection post-cardiac surgery. However, further large multi-centre randomised controlled trials or well-designed prospective trials, which take into account patient and peri-operative factors, standardise the procedure and antibiotic prophylaxis used, and evaluate mupirocin resistance rates, would be invaluable in creating firm guidelines in patients undergoing cardiac surgery to minimise sternal wound infections.
